# eQTLs as causal instruments for the reconstruction of hormone linked gene networks

**DOI:** 10.3389/fendo.2022.949061

**Published:** 2022-08-17

**Authors:** Sean Bankier, Tom Michoel

**Affiliations:** Computational Biology Unit, Department of Informatics, University of Bergen, Bergen, Norway

**Keywords:** causal inference, hormones, genetics, eQTL, networks

## Abstract

Hormones act within in highly dynamic systems and much of the phenotypic response to variation in hormone levels is mediated by changes in gene expression. The increase in the number and power of large genetic association studies has led to the identification of hormone linked genetic variants. However, the biological mechanisms underpinning the majority of these loci are poorly understood. The advent of affordable, high throughput next generation sequencing and readily available transcriptomic databases has shown that many of these genetic variants also associate with variation in gene expression levels as expression Quantitative Trait Loci (eQTLs). In addition to further dissecting complex genetic variation, eQTLs have been applied as tools for causal inference. Many hormone networks are driven by transcription factors, and many of these genes can be linked to eQTLs. In this mini-review, we demonstrate how causal inference and gene networks can be used to describe the impact of hormone linked genetic variation upon the transcriptome within an endocrinology context.

## Introduction

Since the inception of Genome Wide Association Studies (GWAS), nearly two decades ago (1), there has been a steady expansion in the number of studies conducted as well as increases in sample size, yielding a wealth of new genetic associations with complex traits and disease. This approach has offered many new opportunities in endocrinology (2), where hormonal networks are well understood and hence lend themselves to informed mapping approaches. However, loci identified by GWAS alone are insufficient to elucidate the mechanisms by which these traits emerge (3) and efforts to understand the biology underpinning these associations has proved to be a significant challenge.

Much of the genetic research related to hormones has focused on monogenic endocrine disorders with scope for clinical intervention through genetic testing schemes. Examples include Autoimmune Polyglandular Syndrome Type 1 (4) and IPEX syndrome (5) involving germline mutations within the *AIRE* and *FOXP3* genes respectively. However many genetic variants that contribute to different endocrine disorders and risk factors arise from common variants identified from GWAS (2). Hormone measurements have been exploited as GWAS traits to identify regions of the genome associated with hormone levels (**Table 1**), but the downstream consequences of these variants are still poorly understood. However, strides have also been made to link these GWAS hits to transcriptomic variation as expression Quantitative Trait Loci (eQTLs) (6).

**Table 1:** Top 10 GWAS as determined by discovery sample size, obtained from GWAS catalog (7) under EFO term “hormone measurment” (EFO_0004730).

Reported Trait	Discovery sample number	Publication date First author	
Nonatopic asthma or fasting insulin levels	502,660 European	2019-10-24	Zhu Z (37)
IGF-1 measurement	435,516 European	2021-07-05	Barton AR (38)
Total testosterone levels	425,097 European	2020-02-10	Ruth KS (39)
Testosterone levels x smoking behaviour interaction	414,294 European	2021-01-06	Liang X (40)
IGF 1 (Gene-based burden)	409,926 European	2021-10-18	Backman JD (41)
Sex hormone-binding globulin levels	389,354 European	2021-05-12	Martin S (42)
Body mass index and fasting insulin (pairwise)	374,012 European, 16,962 African American or Afro-Caribbean, East Asian, Hispanic or Latin American, South East Asian	2021-02-22	Huang LO (43)
Insulin-like growth factor 1 levels	340,567 European, 5974 African unspecified, 7283 South Asian	2021-01-18	Sinnott-Armstrong N (44)
Circulating leptin levels or HOMA-IR	254,263 Asian unspecified, European, Hispanic or Latin American, NR, South Asian	2020-04-01	Wang X (45)
Fasting insulin	213,645 African American or Afro- Caribbean, East Asian, European, Hispanic or Latin American, South Asian	2021-05-31	Chen J (46)

This mini-review aims to describe how advances in population genetics have helped to identify regions of the genome linked to hormone variation, while highlighting how causal network inference and multi-omic integration can enhance these findings with added biological relevance and context. We first describe how GWAS have been used to identify genetic variants that are associated with complex traits, as well as discussing the limitations of association based approaches. The role of in systems genetics is discussed, including how these have been used to model the impact of genetic variation upon molecular phenotypes. Finally, we describe how causal inference methods have been used to overcome some of the limitations of GWAS and how these can be integrated for the reconstruction of causal molecular networks by using eQTLs as genetic instruments.

## Moving beyond GWAS

GWAS have exploded in popularity over the course of the last decade. In 2022, the NHGRI-EBI GWAS Catalog lists 5690 studies for more than 372,752 genetic associations (7). However most of the genome-wide significant loci identified are of low to moderate penetrance, exacerbating the issue of missing heritability that has been predicted for complex traits (8–11). This includes traits such as type II diabetes where only 10% of heritability is explained by the GWAS variants that have currently been identified (12), although twin and population studies estimate heritability to be between 20-80% (13). Missing heritability, combined with the uniform distribution of GWAS hits across the genome, has even led to speculation of an “omnigenic” model of inheritance in which all genes in trait-related cells play a functional role in the resultant phenotype (8).

The genetic drivers behind many common disease phenotypes present through complex multi-factorial models of inheritance (14), as is the case in instances of obesity (15), cardiovascular disease (16) and type II diabetes (12). The identification of causal Single Nucleotide Polymorphisms (SNPs) is further complicated by the presence of pleiotropy, the phenomenon whereby genetic variation can be seen to influence multiple phenotypic traits (17). Pleiotropy has been shown to be highly prevalent across the human genome (18), with studies showing that up to 90% of trait associated loci are associated with multiple traits (19).

**Figure 1:**
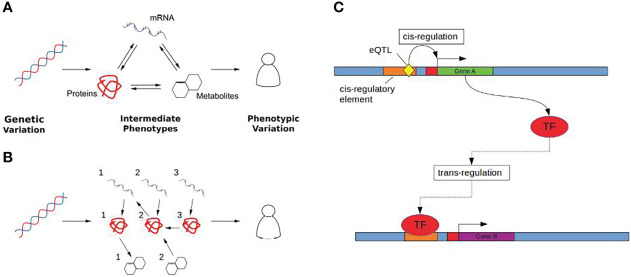
**(A)** Genetic variation (Left) influences complex traits (Right) through quantitative changes in intermediate phenotypes (Middle). Molecular interactions are shown as arrows, where the direction of the arrow indicates the direction of the flow of biological information. **(B)** Intermediate phenotypes can be modelled as biological networks using causal inference to uncover directed relationships between the molecular determinants that mediate the effect of genetic changes on complex traits. **(C)** Cis and trans gene regulation. Gene A (green) encodes a transcription factor (TF) which regulates the expression of gene B (purple). The eQTL (yellow), acts as a cis-eQTL for gene A by causing a change in the sequence of gene A’s cis-regulatory element (orange) which may either increase or decrease the binding affinity of any corresponding TFs. The same eQTL is a trans-eQTL for Gene B as by changing the expression of the TF encoded by gene A, this in turn influences the expression of gene B.

These limitations have encouraged the wider integration of multi-omic data to provide functional context for GWAS results, linking SNPs to intermediate molecular phenotypes using systems genetics approaches that consider the global response to genetic variation (20) (**Figures 1A** **, B**). This is particularly relevant in the case of hormone associated genetic variation, as many hormones mediate signalling across tissues (21) through transcriptional changes and can be modelled using these systems based approaches.

## Retrospective of eQTL studies

As transcriptomic data has become more readily available from highly powered studies, there has been a drive to link SNPs to variation in gene expression. High throughput sequencing technologies such as RNA-sequencing (RNA-seq) have facilitated the analysis of gene expression on a genome wide scale, replacing SNP microarrays as the leading method for gene expression analysis (22). In conjunction with the emergence of large deeply genotyped cohorts, this has allowed for the mapping of eQTLs on an unprecedented scale.

RNA-seq also provides the advantage of allowing for the measurement of Allele-Specific Expression (AES), where it is possible to measure the relative contributions of the maternal and paternal allele, something that is not possible in microarray based methods (23). RNA-seq estimates the expression of different genes through the total read count method, which measures the number of mapped sequence reads (24). This allows for the use of traditional eQTL mapping methods such as linear regression, but also facilitates the direct modelling of gene expression of total read count using discrete distributions (22).

Linking GWAS loci to gene expression provides some indication of a functional relationship, and indeed data have demonstrated that trait-associated SNPs are more likely than non-trait associated SNPs to also be associated with changes in gene expression (eSNPs) (25). eSNPs describe the association between a single SNP with changes in gene expression whereas eQTLs are reflective of the association between a genetic locus and gene expression (26). Although association with changes in gene expression is not a direct proxy for function, this does help to better characterise systemic changes that are elicited in response to trait-associated genetic variation.

eQTLs are categorised on the basis of their proximity to the gene locus with which they are associated (**Figure 1B**). This distinction is important as it provides insight into the mechanisms by which an eQTL mediates an effect on gene expression. Cis-eQTLs, located close to their associated gene, are more likely to be acting locally than those located further away. Typically this distance is defined as being within 1 Mb of the associated transcription start site (27, 28). Outside of this threshold, eQTLs are said to be acting distally with associated genes in trans. Trans-eQTLs can be associated with genes located several megabases away including those on other chromosomes (29).

If an eQTL is cis-acting, this is likely to suggests a physical interaction between the eQTL and the associated gene. For example, a cis-eQTL sitting in an enhancer region may facilitate either an increased or decreased affinity for binding with a transcription factor (30, 31). Trans-eQTLs, on the other hand, associated with a distal gene, may influence transcription indirectly through an intermediary gene product or working in conjunction with local cis-eQTLs (32). Most gene regulation takes place in cis, within regulatory regions and this is reflected by cis signals appearing more strongly than trans effects from eQTL mapping studies (33).

Genomes are consistent between cell types, but the way in which these genes are expressed varies drastically between tissues, and much of the regulation that mediates this disparity takes place at the transcriptome. Tissue gene expression profiles as measured by bulk RNA-seq are composed of distinct cell types, however cellular deconvolution methods can estimate the relative contributions of different cells types within the bulk sample. Methods that use single cell RNA-seq data perform most effectively as described in a recent benchmarking study (34).

Developments and reduced costs for RNA-seq methods has led to the establishment of large multi-tissue eQTL catalogues (**Table 2**). One of the most comprehensive projects to produce an atlas of transcriptome wide genetic effects has been conducted by the GTEx consortium, who have generated tissue specific eQTL data from an impressive number of post-mortem samples. In the latest release of data from GTEx v8 (35), the authors present both trans and cis associations from 49 different tissues. AES methods have also been applied using GTEx with the aim of improving the power of eQTL mapping studies. Zhabotynsky and colleagues show that AES both improves the power to detect eQTLs in GTEx tissues and the quantification of individual-specific genetic effects, while observing a similar levels of enrichment of GWAS hits within eQTL sets as seen using linear models (36).

**Table 2:** Top 10 RNA-seq based eQTL studies as determined by sample size, obtained from eQTL catalogue (47).

Study name	Cell type or tissues	Number of samples	Number of donors
GTEx (v8) (35)	49 tissues	15,178	838
TwinsUK (48)	adipose, LCLs1, skin, blood	1364	433
Schmiedel (49)	15 immune cell types	1331	91
Quach (50)	monocytes	969	200
CommonMind (51)	brain (DLPFC3)	590	590
ROSMAP (52)	brain (DLPFC3)	576	576
GENCORD (53)	LCLs1, fibroblasts, T cells	560	195
FUSION (54)	adipose, muscle	559	302
BLUEPRINT (55)	monocytes, neutrophils, CD4+ T cells	554	197
Nedelec (2016) (56)	macrophages	493	168

## Causal inference in genetic epidemiology

Mendelian randomisation (MR) is a causal inference based approach that has been applied extensively in relation to SNP associated phenotypes (57). During meiosis, alleles are randomly seg- regated within chromosomes during gamete production. This independent assortment ensures that alleles are randomly distributed across a given population, much in the same way that treatments are allocated during randomised controlled trials, hence the “randomisation” in MR refers to the way in which alleles randomly segregate from parent to offspring (58).

**Figure 2:**
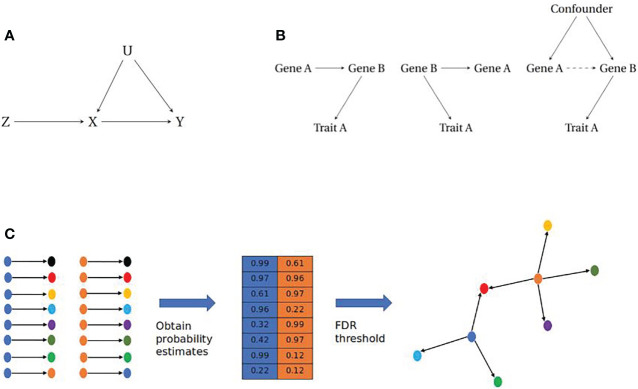
**(A)** Instrumental Variable paradigm. The instrumental variable (Z) is causally associated with the exposure (X) which in turn is causally associated with the outcome (Y). The IV will account for any confounding (U) that affects the exposure or outcome, assuming independence of U. **(B)** Causal modelling of pairwise gene-gene relationships. (Left) Simple causal model where Trait A is influenced by Gene A, through Gene B (Middle) Reactive model where Gene B influences both Gene A and Trait A, therefore any association between Gene A and Trait A is a non-causal relationship. (Right) Association between Genes A and B is a result of unobserved confounding, therefore there is no causal relationship between Gene A and Trait A. **(C)** Reconstructing gene networks from pairwise relationships. (Left) Prospective pairwise relationships between genes with a robust eQTL (blue and orange) and other genes within a dataset. (Middle) Causal inference approaches are employed to obtain a probability matrix for the likelihood of a causal relationship between gene pairs. (Right) A filtering step is imposed e.g. a False Discovery Rate (FDR) cut-off, which will return relationships that cross this threshold to be assembled as directed networks.

MR uses an Instrumental Variable (IV) analysis framework to obtain causal relationships between biological traits (**Figure 2A**). For this methodology, the IV is used to infer a causal relationship between an exposure and an outcome variable. IV analysis requires the following assumptions: 1) The IV should be robustly associated with the exposure. 2) The IV should only be causal for the outcome through the exposure (58). 3) The IV should be independent from any confounding factors that are causal for the exposure or the outcome (59, 60). Given these assumptions, the IV acts as a proxy for the exposure to infer a directed relationship between the exposure and outcome, where the detection of a causal relationship between the instrument and outcome can be inferred as a causal relationship between exposure and outcome, due to the elimination of an alternative causal path. Given the IV assumptions and continuous traits, the average causal effect of the exposure on the outcome can be estimated by the ratio of the covariances of the IV and the exposure and outcome respectively (60).

The use of genetic variants as instruments in MR has become an important method for establishing causal relationships in biological systems where gene expression acts as an exposure. eQTLs have been shown to satisfy the IV assumptions through a robust association with gene expression, given the same eQTL is not also directly associated with the outcome (61). This also overcomes issues related to confounding as genetic variation is fixed at conception and is therefore highly unlikely to be confounded by the same causal factors influencing downstream phenotypes (62) outside of systemic variation in population structure. Issues can arise when an eQTL is also directly associated with the outcome, however this can be overcome through careful instrument selection and testing for pleiotropy (63).

It has been challenging to identify causal variants from GWAS results alone as a result of Linkage Disequilibrium (LD), resulting in the observation of the non-random inheritance of alleles at a given loci with SNPs that are in LD (64). Therefore, if a true causal SNP for a trait is present and detectable at a given locus, the causal SNP and all other SNPs in LD will be identified as being associated with the trait in question, leading to an increase in type I error rate (65). In the instance of using eQTLs to obtain causal estimates between traits, as the eQTL is being used as an instrument it is not necessary that it is causal for the trait in question and eQTLs in LD will also be suitable.

## Reconstruction of causal gene networks

Jansen and Nap first proposed the integration of genomic information to identify changes in continuous molecular traits associated with the segregation of genotypes within a population in 2001 (66). What the authors originally describe as “genetical genomics”, outlines a strategy to link genetic variation within a population to gene expression data, at the time obtained from microarray assays, and to other sources of expression data relating to proteins and metabolites. This has provided the foundation for modern day systems genetics, which allows for the integration of genetic and quantitative data with the ultimate aim of generating biological networks that can be linked to complex traits (20).

Most network based approaches to date have focused on correlation, through the development of co-expression networks using transcriptomic data (67). Co-expression networks were first proposed in the 1990s (68) and have been used to identify novel pathways in complex traits and disease as wide ranging as depression (69), muscular disease (70), and cardiovascular disease (71). They have also been used to identify clusters of genes that are linked to different phenotypic characteristics for conditions such as endometriosis (72). These methods are capable of reconstructing edges (connections between nodes) between co-expressed genes but are limited due to their inability to distinguish between different causal models (**Figure 2B**).

Pairwise gene-gene relationships are capable of providing a foundation for gene network reconstruction using sufficiently large transcriptomic datasets (73–77). MR based methods can be used to obtain probability estimates for causal relationships between genes when provided with robust genetic instruments. A method that facilities this approach is the tool Findr, which incorporates eQTLs within an MR framework to obtain the Bayesian posterior probability of a causal relationship between a pair of genes, using a combination of likelihood ratio tests to account for any unobserved confounding (77).

Bayesian networks are acyclic graphs that have been used for modelling gene networks as they allow for the incorporation of prior knowledge and are capable of resolving issues of conditional independence in data (73, 78–81). Bayesian networks are developed using frequency tables from discrete data, however in cases of continuous data such as transcriptomic datasets, posterior probabilities can be calculated from density functions (82). By obtaining posterior probabilities for pairwise relationships between genes with tools such as Findr, it is possible to reconstruct networks of genes (nodes) that are connected by posterior probabilities (edges) at a given thresh- old (83) (**Figure 2C**).

An issue encountered within Bayesian network analysis, is that as the number of network nodes increases so does the number of potential network edges. Given the high dimensional datasets commonly generated from next generation sequencing, standard Bayesian network methods are often computationally prohibitive (84). Novel methods to overcome the computational burden include the use of eQTL and transcriptomic data within a node ordering approach which prioritises given relationships, reducing the number of possible networks (83).

There is a wealth of data relating to the role of gene regulation, including available cis-regulatory elements (85) and transcription factor binding sites (86). The incorporation of these data allows for the construction of robust priors for Bayesian causal inference (83). eQTLs are also particularly well suited to filling this role and have been used to identify genes driving cardiovascular disease (71), type II diabetes (79) and Acute Myeloid Leukaemia (87) when combined with gene expression data.

A combination of different approaches can be used in the dissection of GWAS hits for complex disease, as demonstrated by Small and colleagues who were able to reconstruct networks of genes associated with SNPs linked to type II diabetes and mediated through the gene *KLF14* (88).

The researchers were able to show that cis-eQTLs for *KLF14* regulated a larger adipose specific gene network that was significantly enriched for metabolic pathways. This highlights the role of a network approach when combined with traditional genetic association and linkage studies. More recently, a 2022 study demonstrated how it was possible to use eQTLs to identify tissue specific clusters driven by “key driver genes” (89). Some of these key drivers were then validated using similar MR based methods as described in this mini-review.

## Conclusion

Over the course of this mini-review we have described how GWAS have been used to link genetic variants to different endocrine traits, including changes in hormone levels. Much of this variation is reflected at the transcriptome, indicated by the presence of cell type specific eQTLs. There have been many thorough studies that have used network based approaches to understand the impact of genetic variation upon phenotypes, although without causal inference, it is challenging to identify the causal drivers of these networks. The use of eQTLs in MR has lead to identification of causal relationships between different traits, including molecular phenotypes. Through an extension of this pairwise approach to causal inference, we propose an systems genetics framework through which the reconstruction of causal gene networks is possible, with particular relevance to endocrinology.

## Author contributions

SB and TM both contributed to the conception and design of this manuscript. The manuscript was written by SB with TM providing supervision, oversight and review. All authors contributed to the article and approved the submitted version.

## Funding

This work has been funded under the Norwegian Research Council (NFR) grant for the project “Intelligent systems for personalized and precise risk prediction and diagnosis of non-communicable diseases” under project number 312045.

## Acknowledgments

The authors would like to acknowledge the assistance of Prof Brian Walker (Newcastle University, UK) and Prof Ruth Andrew (The University of Edinburgh, UK) for contributing to the conception of this manuscript and for the review of early drafts.

## Conflict of interest

The authors declare that the research was conducted in the absence of any commercial or financial relationships that could be construed as a potential conflict of interest.

## Publisher’s note

All claims expressed in this article are solely those of the authors and do not necessarily represent those of their affiliated organizations, or those of the publisher, the editors and the reviewers. Any product that may be evaluated in this article, or claim that may be made by its manufacturer, is not guaranteed or endorsed by the publisher.
